# Chaotic Salp Swarm Optimization-Based Energy-Aware VMP Technique for Cloud Data Centers

**DOI:** 10.1155/2022/4343476

**Published:** 2022-05-12

**Authors:** S. Parthiban, A. Harshavardhan, S. Neelakandan, Vempaty Prashanthi, Abdul-Rasheed Akeji Alhassan Alolo, S. Velmurugan

**Affiliations:** ^1^Department of Computer Science and Engineering, Saveetha School of Engineering, Saveetha Institute of Medical and Technical Sciences, Chennai, India; ^2^Department of Computer Science and Engineering, VNR Vignana Jyothi Institute of Engineering and Technology, Hyderabad, India; ^3^Department of Computer Science and Engineering, R.M.K. Engineering College, Chennai, India; ^4^Department of Marketing and Corporate Strategy, Tamale Technical University, Tamale, Ghana; ^5^Department of Computer Science and Engineering, Vel Tech Multi Tech Dr. Rangarajan Dr. Sakunthala Engineering College, Chennai, India

## Abstract

The amount of energy required by Cloud Data Centers (CDCs) has increased significantly in this digital age, and as a result, there is a pressing need to reduce CDC energy ingesting. Consolidation of virtual machines (VMs) and effective virtual machine placement (VMP) techniques are commonly employed in large data middles to reduce energy consumption. The VMP is an NP-hard subject with infeasible optimum explanations even for tiny data middles, and it is dealt with using the Metaheuristic Optimization Algorithm, which is an experiential approach to optimization. With this in mind, this study introduces a novel energy-aware VMP technique for CDCs that is founded on the Disordered Salp Swarm Optimization Algorithm (EAVMP-CSSA) and is enhanced for energy efficiency (EAVMP-CSSA). The EAVMP-CSSA technique attempts to reduce CDC energy ingesting by dropping the quantity of active servers supporting virtual machines. The recommended EAVMP-CSSA strategy also aims to balance the resource operation of active servers (i.e., CPU, RAM, and Bandwidth), hence reducing waste and increasing efficiency. Furthermore, by combining the ideas of chaotic maps with the standard Salp Swarm Optimization Algorithm (SSA), the CSSA is intended to improve overall performance and reduce computational costs (SSA). A comprehensive range of experimental analyses are performed to ensure that the EAVMP-CSSA technique performs better, and the findings are compared to current VMP techniques. The EAVMP-CSSA approach achieves an effective outcome with a maximum service rate of 98.12%, whereas the Random, FFD, ACO, and AP-ACO procedures achieve a minimum service rate of 74.40%, 78.80%, 90.70%, and 96.31%, respectively. The experimental results demonstrate that the EAVMP-CSSA approach outperforms other assessment metrics.

## 1. Introduction

**Figure 1:**
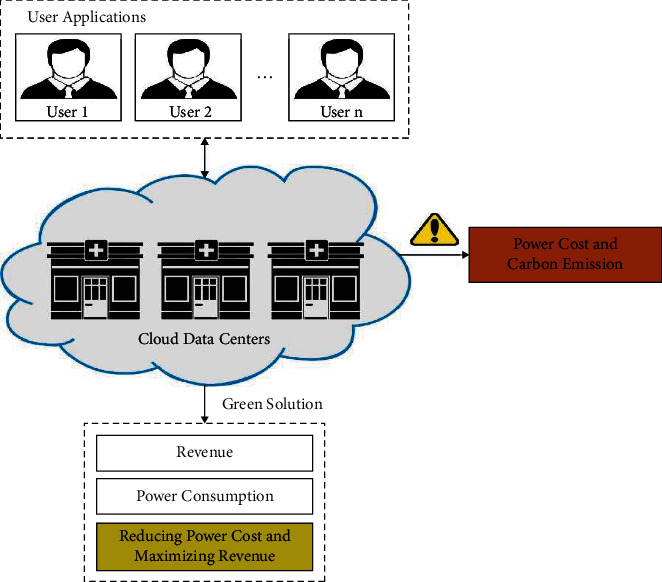
Overview of cloud computing.

Cloud Computing (CC) is one of the effective computing modules that delivers and hosts a broad variety of services via Internet. Several enterprises are based on cloud framework instead of in-house framework for the benefits provided by cloud platforms like removing maintenance burden, attaining on demand scalability, and pay as you go pricing module [1]. The data center is utilized in the cloud platforms for providing cloud services that consumes a huge amount of energy for its processes. An overview of CC perfect is shown in Figure 1. The information center is generally equipped with huge number of physical attendants. Nearly 60% of the overall liveliness ingesting in information midpoint originate from the IT framework that is managed using Physical Machines (PMs). Thus, reducing the number of lively PMs in a data center would greatly enhance the less vigor operation rate. Virtualization is one of the advanced techniques where the CC assets are given to clients through limitless amount of Virtual Machine (VM) depending upon a group of Service Level Agreements (SLAs) among cloud customers and providers [2]. Virtualization performs a critical part in attaining energy efficiency and high server consumption as numerous VMs are assigned to the same corporeal attendant. In a virtualized cloud platform, a sufficient quantity of active attendants is placed based on the VM deployment [3]. Henceforth, utilizing an effective Virtual Machine Placement (VMP) method could attain major impact on a data centers' power utilization.

The VMP problem is similar to the resource allocation problems, concentrating on how to assign physical resources of PM to VM of users with their needs. The main problem in network virtualization is that the VMP problem has attained substantial interest recently. The multidimensional physical resource of the server in data center includes storage resources (Storage), computing resources (CPU), and memory resources (Memory) [4]. To assure the quality of service, it is essential that the server must have adequate multidimensional assets for the application it transports [5]. Through an additional development in transmission methods, it has increasingly different applications [6]. Particularly, memory-intensive, I/O-intensive, and computation-intensive applications coexist on the Internet recently. Apparently, the multiresource requirements vary from application to application. The computationally intensive task is starving for huge CPU prerequisite; however, it demands only small memory.

The VMP method attempts to discover optimum allocations of VM over PM to attain their objectives of the design [7]. Several design objectives are considered in the survey, for example, reducing SLA violation, improving the power utilization, optimizing resource consumption, and so on. The VMP problematic can be considered as an NP-hard optimization problematic. Thus, various metaheuristic methods like Biogeography-Based Optimization (BBO), Glowworm Swarm Optimization (GSO), and Firefly Algorithm (FA) are applied for generating effective solutions within the moderate time [8]. Though, numerous VMP techniques [9, 10] are presented for addressing the energy utilization optimization problem in cloud platforms; they ensure well-adjusted utilization of multidimensional assets amongst dynamic PMs. These methods might allocate distinct number of residual assets for all resource types of PM. In expectation with upcoming demands, the resource left on every PMs must be balanced. Otherwise, the unbalanced residual resources might be avoided by the other VMPs, which leads to wastage of computing resource.

This study designs VMP approach using Chaotic Salp Swarm Optimization Algorithm (EAVMP-CSSA) for Cloud Data Centers (CDCs). The design of CSSA for the optimal placement of VMs shows the novelty of the work. The EAVMP-CSSA technique intends to effectively utilize the energy at the CDCs by reducing the active server count hosting VMs and turning off the idle ones. The planned EAVMP-CSSA technique derives a Fitness Function to achieve minimization of the complete liveliness ingesting of the cloud information servers with minimal resource wastage. Furthermore, it accomplishes the balanced utilization of multiple resources of the active sources to minimalize the resource wastage. To highlight the improved performance of the proposed EAVMP-CSSA technique, a set of simulations are performed and the results are investigated under different aspects.

## 2. Prior Works on VMP Techniques

In Ramalingam and Mohan's work [11], a novel method is presented in accordance with the integration of hybrid optimization algorithms for optimum deployment in CDCs. The initial impartial of the projected method is to decrease the influence utilization of CDCs during the reduction of active PMs. The next aim is to reduce the resource consumption and manage resources by an optimum deployment of VM on PM in the CDC. Nabavi et al. [12] presented a multiobjective VMP system (consider VM as a fog task) for the ECDC is known as TRACTOR that uses an ABC optimization method for energy aware assignments of VM on PM. The projected system aims to reduce the network traffic of the related VMs and energy exploitation during the data center changes and PMs.

Alboaneen et al. [13] planned a novel metaheuristic technique for enhancing Joint Task Scheduling and VMP (JTSVMP) in CDC. The JTSVMP problems consists of two processes, namely, VM placement and task scheduling, that are processed as a combined problematic to be resolved by MOA method. The projected cooptimization procedure purposes to allocate task for the VM that has the minimum performance cost within the fixed limits and later deploy the chosen VM on maximum used PH within capability limit. Gharehpasha et al. [14] projected a novel method with an integration of the SCA and SSA as separate multiobjective and disordered function for a best VMP. The initial purpose of the projected method is to decrease the control utilization in CDCs by reducing the number of lively PMs.

In Abdel-Basset et al.'s work [15], a bandwidth-conscious VMP method has been presented based on the enhanced WOA hybridized by a novel BWAP. The projected study concentrates on improving the bandwidth when the other significant aspects like CPU and memory utilization are not considered. In addition, the power utilization optimization problem was not tackled. At last, Alresheedi et al. [16] presented a cross multiobjective VMP method based on SSA and SCA. The presented method is intended to enhance the SLA violation, MTBHS, and power utilization. The presented method is related to various metaheuristics, and the attained result supports its dominance. But the bandwidth has not been utilized while illustrating PMs and VMs. Additionally, the balanced utilization of multidimensional resources in physical server was not assured.

Wei et al. [17] presented an energy-effective VMP system that continued to decrease the control utilization and transmission cost on circulation conscious datacenter network. For solving this optimization problem, an enhanced ACO using adaptive variable set was proposed for balancing its strong searching ability and fast convergence. Torre et al. [18] presented a multi-impartial technique for active VMP that examines the live relocation method for concurrently improving the overcommitment ratio, migration energy, and resource waste. This optimization method utilizes a new evolution meta-experiential approach founded on the key populace method for approximating the Pareto-optimum set of VMP using better diversity and accuracy.

In Abohamama and Hamouda's work [19], a hybrid VMP method is presented in accordance with permutation-based GA and multidimensional supply conscious best appropriate distribution approach. The projected VMP method purposes at the minimum liveliness utilization amount of cloud datacenters by reducing the number of active servers that hosts VMs. In addition, the suggested VMP method tries to attain balanced application of multidimensional resources (Bandwidth, RAM, and CPU) of lively server that consecutively reduces the resource wastage. Wei et al. [20] balance the multiple resource application for alleviating resource fragmentation when increasing the service rate for VMP, thus avoiding excess of physical resource. For solving this biobjective optimization problem, they presented a joint bin packing heuristic and GA that attains an accurate optimum solution at low time complexity. Reducing the power cost and maintaining the QoS assurance are the two major objectives of this research [2, 18]. To effectively tackle this issue, the presented VM merging method reflects the present and upcoming consumption of possessions by the host Underload Detection (UP-PUD) and host Overload Detection (UP-POD) [19, 21]. The upcoming resource consumptions are precisely forecasted using Gray Markov-based method.

## 3. Background Information and Problem Statement

This section discusses the background details of PMs and VMs. Besides, the problem statement of the proposed model is as follows.

### 3.1. Physical Machines (PMs)

The data center contains *m* PMs *P*={*p*_1_,…, *p*_*m*_}. A resource capacity vector $\overset{⟶}{\text{CV}}\left(J^{2}\right)=\left(c_{1},\ldots ,c_{v}\right)\;$ defines every PM *p* ∈ *P*, whereas each dimension *k* ∈ [1,  *v*] denotes the capability of all PM physical resources *r*_*k*_ in the set *R*={*r*_1_,…, *r*_*v*_}. In a usual Cloud situation, *R*= {*CPU*, *memory*, *disk*, *network*}, abstracted using the virtualization technique [18]. This research emphasizes on memory and CPU, the most committed resource in data center that affects the VM migration [22, 23].

### 3.2. Virtual Machines (VMs)

They recognize dual groups of VMs that contribute to the deployment procedure. The received VMs are the novel VM, which increases the application or generate novel application placements. The hosted VM is now the running one [24, 25]. Together, they determine a set VM={*vm*_1_,…, *vm*_*n*_} deployed on enhanced subsets of PM *P*_used_⊆*P*. All *vm* ∈ VM contains two *v*‐dimensional vectors. *Resource size vector*$\overset{⟶}{\text{SV}}\left(wn\right)=\left(s_{1},\ldots ,s_{v}\right)$ denotes the quantity *Sk* of resource *r*_*k*_ requested by the VM *vm*, using *k* ∈ [1,  *v*], *Resource demand vector*$\overset{⟶}{\text{DV}}\left(vm,\;t\right)=\left(d_{1}\left(t\right),\ldots ,d_{v}\left(t\right)\right)$ determines the *vm* task demands *d*_*k*_(*t*) for every resource *r*_*k*_ at time instance *t*, through *k* ∈ [1, *v*].

### 3.3. Problem Statement

**Figure 2:**
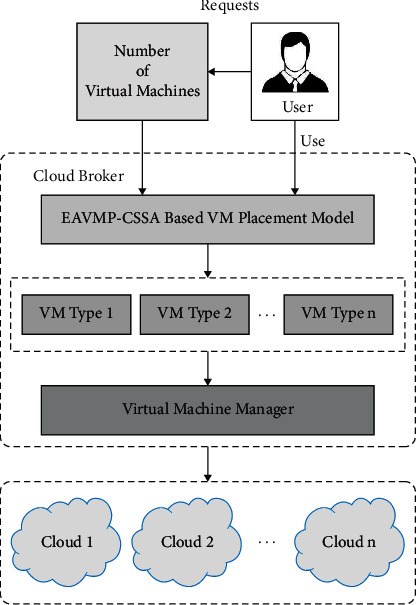
General process involved in VMP.

EAVMP The VSBPP could be summarized as follows: assume an established of inseparable substances by specific masses and established of containers through parameter size (or type), pack the entire items to the quantity of bins; thus, the amount of wasted space of the utilized bin is reduced. In this work, VMs and PHs are signified by the tierce greatest important capitals such as system bandwidth, the CPU, and memory [19, 26]. Given that “*N*” VMs and “*M*” PHs and the overall demand for the VM are lesser compared to the overall capabilities of PH. All VMs should be exactly allocated to one PM ((2) and (4)). All PMs should contain sufficient resources for the allocated VMs ((5)–(7). *ν*_cpu_*i*__, *ν*_mem_*i*__, and *ν*_BW_*i*__ represent the network bandwidth, the CPU, and memory demand of *VM*_*i*_, respectively. *p*_cpu_*j*__, *P*mem_*j*_, and *p*_*Bw*_*f*__ denote the network bandwidth, the CPU, and memory capabilities of PH_*j*_, respectively. The overall procedure of VMP is publicized in Figure 2.

The VMP problems could be equated as VSBPP.

(1) $$\begin{array}{c} \text{Minimize}\sum_{j=1}^{M}c_{j}y_{j}, \end{array}$$

subjected to

(2) $$\begin{array}{c} \chi_{ij}=\left\{\begin{array}{cc} 1, & \text{if server }{\text{PH}}_{j}\text{ is allocated to }VM_{i},\\ 0, & \text{Otherwise,} \end{array}\right) \end{array}$$

(3) $$\begin{array}{c} y_{j}=\left\{\begin{array}{cc} 1, & \text{if }\sum_{i=1}^{N}x_{\text{i}j}\geq 1\\ 0, & \text{Otherwise,} \end{array}\right), \end{array}$$

(4) $$\begin{array}{c} \sum_{j=1}^{M}x_{ij}=1, \end{array}$$

(5) $$\begin{array}{c} \forall j,\;\sum_{i=1}^{N}\nu_{{\text{cpu}}_{i}}\times x_{ij}\leq p_{{\text{cpu}}_{j}}, \end{array}$$

(6) $$\begin{array}{c} \forall j,\;\sum_{i=1}^{N}\nu_{{\text{mem}}_{i}}\times x_{ij}\leq p_{{\text{mem}}_{j}}, \end{array}$$

(7) $$\begin{array}{c} \forall j,\;\sum_{i=1}^{N}\nu_{{\text{BW}}_{i}}\times x_{ij}\leq p_{Bw_{j}}. \end{array}$$

Here, *y*_*j*_ denotes the binary parameter that specifies whether PH_*j*_ has VMs or not, *c*_*j*_ represents the cost/wasted space of PM PH_*j*_, *x*_*ij*_ represents the binary parameter that specifies whether VM_*i*_ is allocated to PH_*j*_ or not, *N* indicates the overall VMs, and *M* represents the overall PMs, *i* ∈ {1,  2,…, *N*} and *j* ∈ {1,  2,…, *M*}.

## 4. Design of EAVMP-CSSA Technique

SSA is a recently presented metaheuristic algorithm that inspires the behaviour of salps in ocean. It is a class of Salpidae similar to that of jelly fish. It forages as well as navigates in a swarm that represents salp chain. SSA is a new kind of PSO that modules the salp cable [21, 27]. The salp populace has follower and leader salps. The location of every salp is in *d* dimension search space, where *d* denotes the quantity of parameters in a specific problematic, like additional group-built method. The present location course of *n* salp in the exploration interplanetary is *X*^*j*^=[*x*_1_^*j*^, *x*_2_^*j*^,  *x*_3_^*j*^,…, *x*_*d*_^*j*^], *j*=1,2,…, *n*. The spearhead salp upgrades its location, and it is given by

(8) $$\begin{array}{c} X_{i}^{1}=\left\{\begin{array}{cc} F_{i}+C_{1}\left(\left(ub_{i}-ul_{i}\right)C_{2}+lb_{i}\right), & C_{3}\geq 0,\\ F_{i}-C_{1}\left(\left(ub_{i}-ul_{i}\right)C_{2}+lb_{i}\right), & C_{3}<0, \end{array}\right) \end{array}$$

where *X*_*i*_^1^ denotes the location of the spearhead salp in the *i*^*th*^ measurement, *F*_*i*_ represents food location in the *i*^*th*^ measurement, and *ub*_*i*_ and *lb*_*i*_ characterize higher and lesser boundaries in the *i*^*th*^ measurement correspondingly. *C*_1_, *C*_2_, and *C*_3_ denote module coefficients. These coefficients are arbitrary values that are utilized for specific determinations [26, 27]. The initial coefficient *C*_1_ represents the balance between exploitation and exploration that denotes the primary variable in the method. *C*_1_ is determined by

(9) $$\begin{array}{c} C_{1}=2e^{-{\left(4t/{\text{T}}_{\text{max}}\right)}^{2}}, \end{array}$$

where *t* denotes the present repetition and *T*_max_ indicates the extreme number of repetitions. *C*_2_ and *C*_3_ represent arbitrary values created uniformly that lies between zero and one. The follower salp updates their position based on Newton's law of motion, and it is given by

(10) $$\begin{array}{c} X_{i}^{k}=\frac{1}{2}\left(X_{j}^{k}+X_{\text{i}}^{k-1}\right)2\leq k<n, \end{array}$$

where *X*_*i*_^*k*^ denotes the location of *kth* supporter salp in the *i*^*th*^ dimension and *n* represents the entire amount of salp subdivisions. The process involved in SSA is given in Algorithm 1. Population-based metaheuristic method shares different benefits that includes simplicity, scalability, and computation time reduction. But this method has two major drawbacks, namely, low convergence rate and recession in local optimal. A specific method to conquer this problem and improve the efficacy of meta experiential procedures is to place the disorder model. The disordered chart is applied rather than arbitrary values in PSO-based method for enhancing the convergence.

In this technique, the present chaotic-based SSA (CSSA) substitutes arbitrary variable quantity with disordered ones. CSSA utilizes chaotic map for adjusting the values of succeeding constant *C*_2_. The value of *C*_2_ could be substituted by the value of a suitable disordered chart at the present repetition, and it is given by

(11) $$\begin{array}{c} C_{2}^{t}=\omega \left(t\right), \end{array}$$

where *ω*(*t*) denotes the rate of disordered chart at *t*^*th*^ repetition. Equation (8) could be rephrased with the novel rate of *C*_2_, and it is given by

(12) $$\begin{array}{c} X_{i}^{1}=\left\{\begin{array}{cc} F_{i}+C_{1}\left(\left(ub_{i}-ul_{i}\right)\omega \left(r\right)+lb_{i}\right), & C_{3}\geq 0,\\ F_{i}-C_{1}\left(\left(ub_{i}-ul_{i}\right)\omega \left(r\right)+lb_{i}\right), & C_{3}<0. \end{array}\right) \end{array}$$

The chaos model is a popular numerical method utilized for analyzing the behavior of dynamic systems using crucial primary conditions. The specific method to show this behavior by utilizing chaotic map is moreover separate or else incessant. Disordered charts could be placed only for deterministic organizations using prediction performance. At present, confusion model turns more interesting in many streams like robotics, computer science, microbiology, and physics. The chaotic map becomes the robust solution for enhancing the efficiency of metaheuristic method with the enhancement of their arbitrary variables [27–29]. This arbitrary parameter is extracted on the basis of unchanging or Gaussian delivery and hence they could be managed better by using the chaotic maps that share the similar characteristic through higher efficiency. Manage this parameter using a chaotic chart, and the local optimal is reduced, whereas the meeting is increased. The logistic chart is the optimal disordered chart for this optimizer based on the outcome of the optimization. Robert first presented the logistic chart on May 1, 1976, and it has been in use ever since. The following is the most commonly used formula for a logistic disordered map:

(13) $$\begin{array}{c} \omega \left(t+1\right)=a\omega \left(t\right)\left[1-\omega \left(t\right)\right],\;a=4, \end{array}$$

where *ω*(*t*) means the rate of disordered chart at *t*^*th*^ repetition. The original disorder of the disordered charts is considered to be 0.7 (*ω*(0)=0.7).

Provided “*N*” VMs and “*M*” PMs, the EAVMP-CSSA technique recommends various variations for the VMs, which is required to be allocated to the existing PMs. The main objective of the EAVMP-CSSA technique is to decrease the total liveliness operation of the used PMs and thereby minimize the total cost of the cloud provider. The fitness function is given as follows.

(14) $$\begin{array}{c} \text{Minimize }f\left(x\right)=\left[\sum_{j=1}^{M}y_{j}\times \left(\left(P_{j}^{\text{busy}}-P_{j}^{\text{idle}}\right)\times U_{j}^{\text{cpu}}+P_{j}^{\text{idle}}\right)\right], \end{array}$$

where *f*(*x*) signifies the entire liveliness utilization of the PMs, *y*_*j*_ is a binary mutable that designates whether PH_*j*_ comprises VMs or not, *P*_*j*_^busy^ is the higher energy operation of PM PH_*j*_, *P*_*j*_^idle^ is the lower energy utilization of PM PH_*j*_(*P*_*j*_^idle^ ≈ 0.6*∗P*_*j*_^busy^), and *U*_*j*_^cpu^ is the CPU operation ratio of PM PH_*j*_, and it is given by

(15) $$\begin{array}{c} U_{j}^{\text{cpu}}=\frac{\sum_{i=1}^{N}x_{ij}\times \nu_{{\text{cpu}}_{i}}}{p_{{\text{cpu}}_{j}}}, \end{array}$$

where *x*_*ij*_ is the binary parameter indicating whether VM_*i*_ is allocated to PH_*j*_ or not, *ν*_cpu_i__ is the CPU demand of VM VM_*i*_, and *p*_cpu_*j*__ is the CPU volume of PM PH_*j*_.

Provided a VM which is to be allocated, the EAVMP-CSSA technique selects a PM which offers the capitals (CPU, memory, and system bandwidth) required by the VMs [7, 30–32]. The EAVMP-CSSA technique chooses the PM with low-resource wastage after allocating to the present VM. In order to completely exploit the multidimensional resources, the following equation is used to determine the wasted resources.

(16) $$\begin{array}{c} W_{j}=\frac{\left|2L_{j}^{\text{cpu}}-L_{j}^{\text{mem}}-L_{j}^{BW}\right|+\varepsilon }{U_{j}^{\text{cpu}}+U_{j}^{\text{mem}}+U_{j}^{\text{BW}}}, \end{array}$$

where *W*_*j*_ denotes the resource wastage of PM *PH*_*j*_.*L*_*j*_^cpu^, *L*_*j*_^mem^, and *L*_*j*_^BW^ represent the normalized residual CPU, memory, and bandwidth of PM PH_*j*_, respectively. *U*_*j*_^cpu^, *U*_*j*_^mem^, and *U*_*j*_^BW^ represent the regularized CPU, reminiscence, and bandwidth utilization of PM PH_*j*_ correspondingly [33]. *ε* is an actual unimportant hopeful real number and the value is fixed to 0.0001. The aim of (16) is to successfully utilize the multidimensional resources and stability the residual possessions on every PM along distinct extent. *U*_*j*_^cpu^ is already represented in (15), while the rest of the rapports are represented as follows:

(17) $$\begin{array}{c} L_{j}^{\text{cpu}}=\frac{p_{{\text{cpu}}_{j}}-\left(\sum_{i=1}^{N}x_{ij}\times \nu_{{\text{cpu}}_{i}}\right)}{p_{{\text{cpu}}_{j}}},\\ L_{j}^{\text{mem}}=\frac{p_{{\text{mem}}_{j}}-\left(\sum_{i=1}^{N}x_{ij}\times \nu_{{\text{mem}}_{i}}\right)}{p_{\text{mem}m_{j}}},\\ L_{j}^{\text{BW}}=\frac{p_{{\text{BW}}_{J}}-\left(\sum_{i=1}^{N}x_{ij}\times \nu_{{\text{BW}}_{i}}\right)}{p_{{\text{BW}}_{j}}},\\ U_{j}^{\text{mem}}=\frac{\sum_{i=1}^{N}x_{ij}\times \nu_{{\text{mem}}_{i}}}{p_{{\text{mem}}_{j}}},\\ U_{j}^{\text{BW}}=\frac{\sum_{i=1}^{N}x_{ij}\times \nu_{{\text{BW}}_{i}}}{p_{{\text{BW}}_{j}}}, \end{array}$$

where *ν*_cpu_*i*__, *ν*_mem_*i*__, and *ν*_BW_*i*__ signify the CPU, reminiscence, and system bandwidth anxieties of *VM*_*j*_ correspondingly. *p*_cpu_*j*__, *p*_mem_*j*__, and *p*_BW_*j*__ signify the CPU, reminiscence, and network bandwidth dimensions of PH_*j*_ correspondingly. *x*_*ij*_ is a binary adjustable representing whether VM_*j*_ is allocated to PH_*j*_ or not.

## 5. Performance Validation

**Figure 3:**
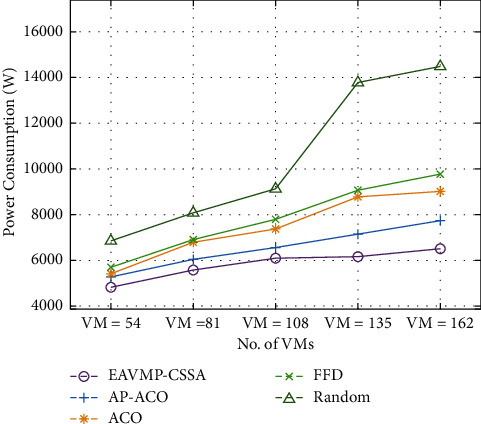
Comparative result analysis of EAVMP-CSSA technique in terms of power consumption.

This unit deals with the presentation analysis of the EAVMP-CSSA method with other prevailing methods in terms of different evaluation parameters.  Table 1 and Figure 3 investigate the power consumption examination of the EAVMP-CSSA method with other methods under varying VMs. The experimental result highlights that the EAVMP-CSSA technique has attained a minimal power consumption over the other methods under distinct VMs.

For instance, with 54 VMs, the EAVMP-CSSA technique has attained a reduced power consumption of 4817 W whereas the AP-ACO, ACO, FFD, and Random techniques have achieved an increased power consumption of 5283 W, 5399 W, 5690 W, and 6853 W, respectively. Moreover, with 108 VMs, the EAVMP-CSSA technique has resulted a lower power consumption of 6097 W whereas the AP-ACO, ACO, FFD, and Random techniques have attained a higher power consumption of 6562 W, 7376 W, 7783 W, and 9121 W, respectively. Furthermore, with 162 VMs, the EAVMP-CSSA technique has attained a reduced power consumption of 6504 W whereas the AP-ACO, ACO, FFD, and Random techniques have achieved an increased power consumption of 7725 W, 9005 W, 9761 W, and 14472 W, respectively.

**Figure 4:**
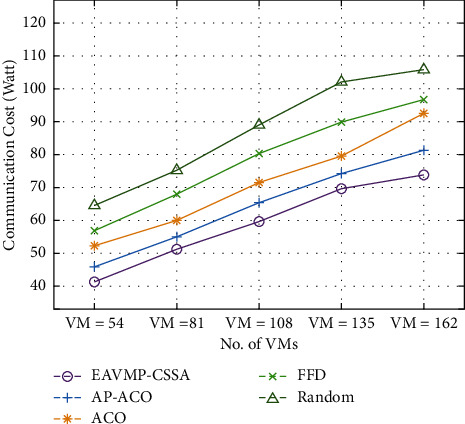
Comparative result analysis of EAVMP-CSSA technique in terms of communication cost.

Table 2 and Figure 4 examine the communication cost examination of the EAVMP-CSSA method with other methods under varying VMs. The simulation values pointed out that the EAVMP-CSSA technique has gained the least communication cost over the other methods underneath variable VMs. For occurrence, with 54 VMs, the EAVMP-CSSA method has an effective outcome with the least communication cost of 41.24 W whereas the AP-ACO, ACO, FFD, and Random techniques have accomplished a higher communication cost of 45.83 W, 52.32 W, 56.91 W, and 64.55 W, respectively.

Eventually, with 108 VMs, the EAVMP-CSSA technique has gained a reduced communication cost of 59.58 W whereas the AP-ACO, ACO, FFD, and Random techniques have resulted an increased communication cost of 65.32 W, 71.43 W, 80.22 W, and 89.01 W, respectively. Meanwhile, with 162 VMs, the EAVMP-CSSA technique has demonstrated better performance with the minimal communication cost of 73.72 W whereas the AP-ACO, ACO, FFD, and Random techniques have accomplished a maximum communication cost of 81.36 W, 92.44 W, 96.67 W, and 105.79 W, respectively.

**Figure 5:**
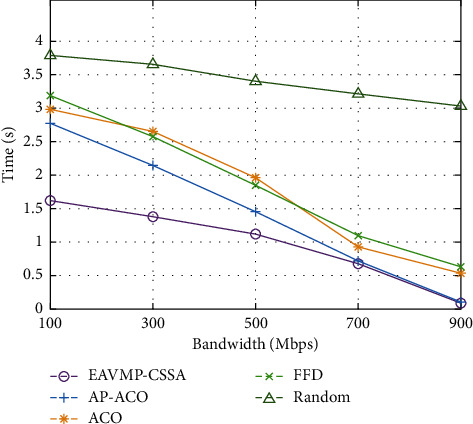
Comparative result analysis of EAVMP-CSSA technique in terms of running time.

Table 3 and Figure 5 assess the time examination of the EAVMP-CSSA method with other methods under varying bandwidth. The found simulation result depicts that the EAVMP-CSSA method has occasioned with the larger presentation with minimum time over the other approaches under varying bandwidth. For instance, under 100 Mbps bandwidth, the EAVMP-CSSA technique has portrayed effectual performance with the lesser time of 1.62 s whereas the AP-ACO, ACO, FFD, and Random techniques have depicted a higher time of 2.77 s, 2.98 s, 3.18 s, and 3.78 s, respectively.

Additionally, with 500 Mbps bandwidth, the EAVMP-CSSA technique has demonstrated with the lesser time of 1.12 s whereas the AP-ACO, ACO, FFD, and Random techniques have taken an increased time of 1.45 s, 1.96 s, 1.85, and 3.40 s, respectively. At last, with 900 Mbps bandwidth, the EAVMP-CSSA technique has gained an optimal outcome with the minimal time of 0.09 s whereas the AP-ACO, ACO, FFD, and Random techniques have accomplished with the maximum time of 0.11 s, 0.54 s, 0.63 s, and 3.03 s, respectively.

**Figure 6:**
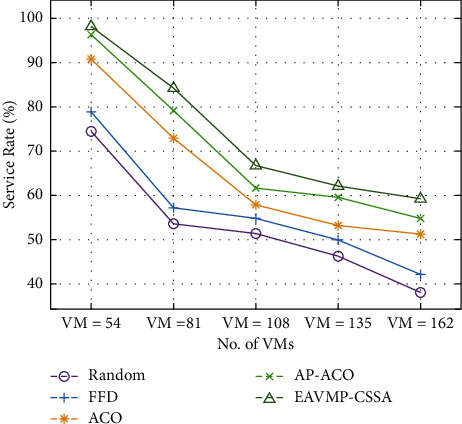
Comparative result analysis of EAVMP-CSSA technique in terms of service rate.

Finally, a service rate examination of the EAVMP-CSSA method takes residence beneath varying number of VMs in Table 4 and Figure 6. The figure proves that the EAVMP-CSSA system has outperformed over the other methods with the maximum service rate for all the VMs. For instance, with 54 VMs, the EAVMP-CSSA technique attains an effectual outcome with the maximum service rate of 98.12% whereas the Random, FFD, ACO, and AP-ACO techniques have gained a minimum service rate of 74.40%, 78.80%, 90.70%, and 96.31%, respectively.

In the same way, with the presence of 162 VMs, the EAVMP-CSSA technique has illustrated proficient performance with an increased service rate of 59.20% whereas the Random, FFD, ACO, and AP-ACO methods have caused in the abridged service rate of 38%, 42.10%, 51.20%, and 54.80%, respectively. From the above benches and statistics, it is obvious that the EAVMP-CSSA method is originate to be an effective instrument for VMP in CDCs.

## 6. Conclusion

This paper has designed a novel EAVMP-CSSA method to achieve liveliness competence in CDCs. The EAVMP-CSSA method is mainly based on the design of CSSA with the integration of chaotic maps and conventional SSA. In addition, the EAVMP-CSSA technique derives an objective function to reduce energy utilization and resource wastage (e.g., CPU, RAM, and bandwidth). The proposed model has the ability to reduce the active server count by balancing the active servers that enables them to accommodate the upcoming VMP requests and eliminates the requirement of activating other servers. The performance of the EAVMP-CSSA technique is examined by the CloudSim tool and the results are investigated under different dimensions. The simulation results confirmed the betterment of the proposed EAVMP-CSSA technique over the recent state of art techniques. EAVMP-CSSA technique attains an effectual outcome with the maximum service rate of 98.12% whereas the Random, FFD, ACO, and AP-ACO techniques have gained a minimum service rate of 74.40%, 78.80%, 90.70%, and 96.31%, respectively. In the future, the design of EAVMP-CSSA technique can be extended to the design of task scheduling techniques to allocate resources in an optimal way. Besides, the presented technique can be employed to eradicate the overutilization of resources, which degrades the VM performance.

## Figures and Tables

**Algorithm 1:**
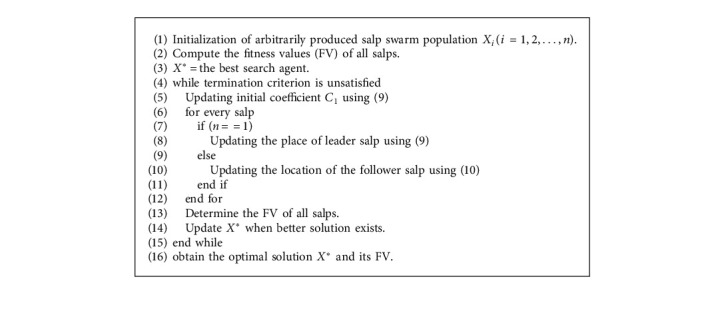
Pseudocode of SSA.

**Table 1:** Power consumption analysis of EAVMP-CSSA technique under varying VMs.

Power consumption (W)					
No. of VMs	EAVMP-CSSA	AP-ACO	ACO	FFD	Random

VM = 54	4817	5283	5399	5690	6853
VM = 81	5573	6039	6795	6911	8074
VM = 108	6097	6562	7376	7783	9121
VM = 135	6155	7144	8772	9063	13774
VM = 162	6504	7725	9005	9761	14472

**Table 2:** Communication cost analysis of EAVMP-CSSA technique under varying VMs.

Communication cost (watt)					
No. of VMs	EAVMP-CSSA	AP-ACO	ACO	FFD	Random

VM = 54	41.24	45.83	52.32	56.91	64.55
VM = 81	51.18	55.00	59.97	67.99	75.25
VM = 108	59.58	65.32	71.43	80.22	89.01
VM = 135	69.52	74.10	79.45	89.77	102.00
VM = 162	73.72	81.36	92.44	96.67	105.79

**Table 3:** Running time analysis of EAVMP-CSSA technique under varying bandwidth.

Time (s)					
Bandwidth (Mbps)	EAVMP-CSSA	AP-ACO	ACO	FFD	Random

100	1.62	2.77	2.98	3.18	3.78
300	1.38	2.14	2.65	2.57	3.65
500	1.12	1.45	1.96	1.85	3.40
700	0.68	0.72	0.93	1.10	3.21
900	0.09	0.11	0.54	0.63	3.03

**Table 4:** Service rate analysis of EAVMP-CSSA technique under varying VMs.

Service rate (%)					
No. of VMs	Random	FFD	ACO	AP-ACO	EAVMP-CSSA
VM = 54	74.40	78.80	90.70	96.31	98.12
VM = 81	53.54	57.10	72.90	79.10	84.20
VM = 108	51.30	54.80	57.80	61.60	66.70
VM = 135	46.20	49.90	53.20	59.50	62.10
VM = 162	38.00	42.10	51.20	54.80	59.20

## Data Availability

The manuscript contains all of the data.
